# Antimalarial Activity of the Chemical Constituents of the Leaf Latex of *Aloe pulcherrima* Gilbert and Sebsebe

**DOI:** 10.3390/molecules21111415

**Published:** 2016-10-28

**Authors:** Tekleab Teka, Daniel Bisrat, Mariamawit Yonathan Yeshak, Kaleab Asres

**Affiliations:** 1 Department of Pharmacy, College of Health Sciences, Wollo University, P.O. Box 1145, Dessie, Ethiopia; ttekleab@gmail.com; 2 Department of Pharmaceutical Chemistry and Pharmacognosy, School of Pharmacy, College of Health Sciences, Addis Ababa University, Addis Ababa, Ethiopia; daniel.bisrat@aau.edu.et (D.B.); mariamawit.yonathan@aau.edu.et (M.Y.Y.)

**Keywords:** *Aloe pulcherrima*, antimalarial, nataloin, 7-hydroxyaloin, four-day suppressive

## Abstract

Malaria is one of the three major global public health threats due to a wide spread resistance of the parasites to the standard antimalarial drugs. Considering this growing problem, the ethnomedicinal approach in the search for new antimalarial drugs from plant sources has proven to be more effective and inexpensive. The leaves of *Aloe pulcherrima* Gilbert and Sebsebe, an endemic Ethiopian plant, are locally used for the treatment of malaria and other infectious diseases. Application of the leaf latex of *A. pulcherrima* on preparative silica gel TLC led to the isolation of two *C*-glycosylated anthrones, identified as nataloin (**1**) and 7-hydroxyaloin (**2**) by spectroscopic techniques (UV, IR, ^1^H- and ^13^C-NMR, HR-ESIMS). Both the latex and isolated compounds displayed antimalarial activity in a dose-independent manner using a four-day suppressive test, with the highest percent suppression of 56.2% achieved at 200 mg/kg/day for **2**. The results indicate that both the leaf latex of *A. pulcherrima* and its two major constituents are endowed with antiplasmodial activities, which support the traditional use of the leaves of the plant for the treatment of malaria.

## 1. Introduction

Malaria is a parasitic disease caused by protozoa species of the *Plasmodium* genus, and it is transmitted to humans through the bite of infected female mosquitoes of the *Anopheles* genus [1]. It is one of the major global public health threats, having a greater morbidity and mortality than any other infectious diseases of the world [2,3]. It is estimated that there are half a billion clinical cases with a corresponding mortality rate of 2 to 3 million annually, 90% of these being in the Sub-Saharan African region, particularly children under five years of age [4,5]. The emergence of drug-resistant parasites, changes in climatic conditions over a large part of Africa, changes in land use and human migration are some of the major factors exacerbating the malaria threat in Africa [6]. Recently, much attention has been given to medicinal plants to offer new and novel antimalarial agents.

In Sub-Saharan African region, hundreds of plants are traditionally used for the treatment of malaria, and many of these plants are used as a part of folk medicine [7]. Ethnobotanical surveys conducted on antimalarial plants are readily available in the literature [8,9,10,11,12]. However, scientific studies aimed at validating the genuine antiplasmodial activities of these plants and elucidating the structures of their active chemical constituents, which are necessary for new drug development, are very limited. According to a survey conducted on medicinal plants traditionally used for the treatment of malaria in eastern Ethiopia, *Aloe* species are among the most commonly-reported plants [9]. Other reports also indicate that the leaf latex of several *Aloe* species possesses genuine antiplasmodial activity [13,14,15,16]. Methods of the evaluation of the antimalarial activity of plant derivatives that rely on the in vivo treatment of rodents infected with *P. berghei* provide insights into the pharmacokinetics and immunological factors, but use non-human malaria parasites [17]. Although direct extrapolation from rodent model biology to *P. falciparum* biology might not be applicable in all situations, the rodent malaria-causing *P. berghei* parasites have been reported to have similar characteristics as *P. falciparum*, making them suitable for a parallel study. For example, similar in vivo drug testing results [18] and the development of cerebral malaria [19] with *P. berghei* have been noted.

**Figure 1:**
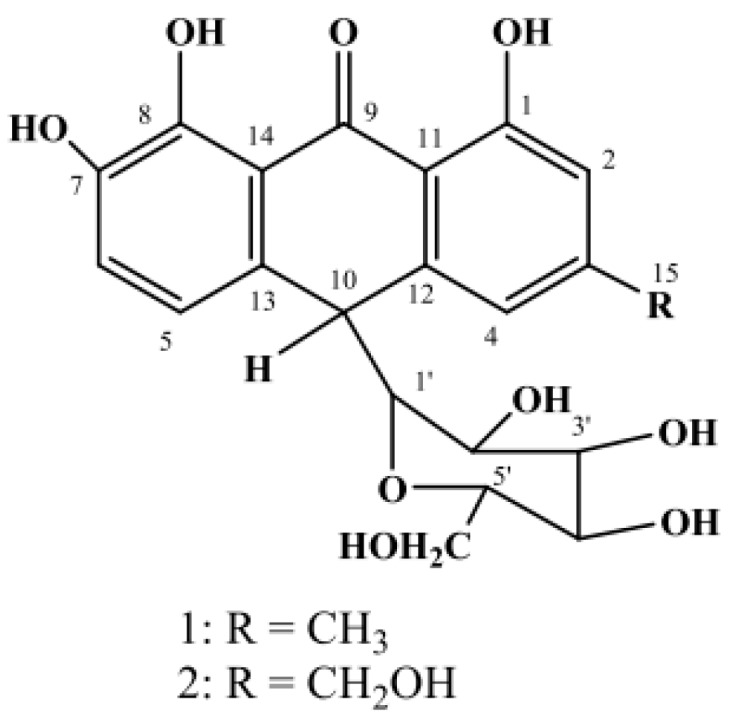
Chemical structures of nataloin (**1**) and 7-hydroxyaloin (**2**).

In view of the above, the leaf latex of *A. pulcherrima*, an endemic *Aloe* species of Ethiopia traditionally used for the treatment of malaria and many other infectious diseases [20,21], was investigated for its in vivo antimalarial activity using mice infected with *P. berghei* parasites in a four-day suppressive test. Furthermore, the present study examined the antiplasmodial activity of two major anthrone glycosides, namely nataloin (**1**) and 7-hydroxyaloin (**2**) (Figure 1), isolated from the leaf latex of *A. pulcherrima*. Although several anthraquinones, naphthoquinones and benzoquinones are known to have genuine antiplasmodial activity [22], only a few anthrones have been reported to have activity against the malaria parasites [23,24].

## 2. Results and Discussion

### 2.1. Isolation and Structure Elucidation of Compounds ***1*** and ***2***

The latex of *A. pulcherrima* was prepared by cutting the leaf transversely close to the stem and inclining the leaf so that the latex flows out for a few hours. Compounds **1** and **2** (Figure 1) were isolated from the dried latex by preparative TLC and characterized on the basis of their spectroscopic properties and by comparison with literature values [25]. 

Compound **1** was obtained as a pale yellow solid amorphous substance with an *R_f_* value of 0.54 in CHCl_3_/CH_3_OH at a 4:1 ratio. It showed a pseudomolecular ion at *m*/*z* of 417.11879 [M − H]^−^ in the negative-mode HRESI-mass spectrum (exact calcd. mass 417.118560 [M − H]^−^) corresponding to a molecular formula of C_21_H_22_O_9_. The presence of an anthrone skeleton was deduced from the UV spectrum (λ_max_: 267, 275 and 309 nm), ^1^H- and ^13^C-NMR spectral data. As clearly shown in the NMR data (see the Experimental Section), Compound **1** was unequivocally identified as 10-*C*-β-d-glucopyranosyl-1,7,8-trihydroxy-3-methyl-10*H*-anthracen-9-one, commonly known as nataloin (Figure 1), by comparing its ^1^H- and ^13^C-NMR data with those reported for the same compound in the literature [25].

Compound **2** was also isolated as a pale yellow solid amorphous substance with an *R_f_* value of 0.34 in CHCl_3_/CH_3_OH (4:1). Analysis of Compound **2** by high resolution negative ion electrospray ionization-mass spectrometry gave a pseudomolecular ion at *m*/*z* of 433.11357, corresponding to a molecular formula of C_21_H_22_O_10_ (exact calcd. mass 433.113475 [M − H]^−^). It was identified as an anthrone derivative from the UV spectrum (λ_max_: 266, 274 and 307 nm), ^1^H- and ^13^C-NMR spectral data [25]. Analysis of the ^1^H-NMR, ^13^C-NMR and DEPT-135 NMR of Compound **2** showed similar data to that of Compound **1**, except the replacement of a methyl group in **1** by an oxymethylene group in **2**. Hence, Compound **2** was unambiguously characterized as 10-*C*-β-d-glucopyranosyl-1,7,8-trihydroxy-3-(hydroxymethyl)-10*H*-anthracen-9-one, commonly known as 7-hydroxyaloin (Figure 1), by comparing its ^1^H- and ^13^C-NMR spectra data with those reported for the same compound in the literature [25].

### 2.2. Acute Toxicity Activity

Acute toxicity studies indicated that although no mortality in both the latex (5 g/kg) and isolated compounds (2 g/kg) was recorded within 14 days, some signs of toxicity, such as decreased locomotion, intermittent diarrhea, abdominal distension, hair erection and loss of appetite, were observed in the first three days. However, as the days went by, these signs of toxicity gradually disappeared. This result shows that the LD_50_s of the latex and the isolated compounds was beyond 5000 mg/kg and 2000 mg/kg body weight, respectively. Weak signs of toxicity displayed by the latex could be due to its major constituents, nataloin and 7-hydroxyaloin, as both compounds showed some signs of toxicity. This finding is consistent with the report from Matsuda et al. [26], who reported that rats fed with *Aloe arborescens* Miller var. *natalensis* Berger up to a dose of 0.16% of their diet for a period of twelve months showed minor adverse effects, which lasted for a short period of time. Thus, the experimental determination of the lack of acute toxicity at higher doses would justify the use of the plant extract for malaria treatment.

### 2.3. In Vivo Antimalarial Activity

In the present study, the parasitemia of *Plasmodium berghei-*infected mice was established as indicated by the level of parasitemia in the untreated group. There was a significant decrease (*p* < 0.05) in the parasitemia of test substance-treated groups, particularly those treated with a dose of 200 mg/kg (Table 1). Furthermore, a significant decrease (*p* < 0.05) of parasitemia was observed in groups treated with 400 mg/kg of the test substances with the exception of 7-hydroxyaloin (**2**), when compared to the untreated group. The percentage of chemosuppression was determined as the percentage of the average parasitemia relative to the negative control. Maximum percentage chemosuppression was obtained for 7-hydroxyaloin (**2**), which showed 56.2% suppression at a dose of 200 mg/kg. The chloroquine-treated group gave a percentage chemo-suppression value of 100%.

It is worthy to note that both the latex and isolated compounds exhibited a reduction in parasitemia with a corresponding increase in chemosuppression as the dose increased from 100 to 200 mg/kg, indicative of antimalarial activity [27]. However, a further increase of the dose of the test substances to 400 mg/kg resulted in the increase of the percentage parasitemia with a corresponding reduction of chemosuppression, indicative of reduced antimalarial activity. Similar results were obtained by Maje et al. [28], whereby the middle dose of the ethanolic leaf extract of *Paullinia pinnata* showed a higher chemosuppressive effect than the upper and lower doses. 

Comparison among the test substance dose levels revealed that 200 mg/kg body weight significantly prolonged (*p* < 0.05) survival time when compared to the negative control. In the four-day suppressive test, administration of the pure compounds to mice at all dose levels did not prevent body weight loss when compared to the negative control (Table 2). However, animals treated with the lowest dose (100 mg/kg) of the latex and those treated with chloroquine prevented body weight reduction significantly (*p* < 0.05) when compared to the negative control. The observed body weight loss in the experimental animals could be due to the appetite-suppressing effect of the test substances, which is in agreement with previous studies carried out on other medicinal plants [29,30]. The fact that both the pure compounds and latex produced the highest parasitemia suppression at the middle dose suggested that 200 mg/kg body weight might be the optimal therapeutic dose in mice. As expected, the 200 mg/kg body weight of all of the test substances prolonged the mean survival time of the study mice indicating that it suppressed *P. berghei* and reduced the overall pathologic effect of the parasite on the study mice.

The mechanism of action of the main components of the latex of *A. pulcherrima* needs to be elucidated. However, plausible mechanisms by which the compounds exerted their action could be proposed based on their structural features. Perusal of the literature reveals that some flavonoid derivatives, xanthones, stilbenes, coumarins, lignans, tannins, quinones, terpenoids, steroids and alkaloids possess antimalarial activity [31]. Among these, natural, semi-synthetic and synthetic quinones have been shown to be effective antimalarials. The mechanism of action of quinones is mostly related to their ability to undergo redox cycling [32]. For example, atovaqone, which is an effective antimalarial drug against the multidrug-resistant parasite [22], acts by inhibiting the mitochondrial electron transport chain [33]. It was also demonstrated that semi-synthetic thiazinoquinones exert antiplasmodial activity by interacting with Fe(III)-protoporphyrin IX [32]. Recently, some natural quinones have been shown to have antimalarial action through inhibition of enzymes. Such compounds include the halenaquinone-type polyketides halenaquinone 1 and orhalquinone 8, which were shown to inhibit phospholipase A_2_ and farnesyltransferase, respectively [34]. Orhalquinone 8 possesses a novel tetracyclic structure with a promising scaffold from which more potent and selective inhibitors of farnesyltransferase could be developed. The schizonticidal effect of anthraquinones is attributed to their cyclic planar structure, which intercalates with parasite DNA [35]. Naphthoquinones and anthraquinones have structural similarity with the isolated compounds. 

## 3. Experimental Section

### 3.1. General Procedures

IR spectra were carried out in KBr plates on a Perkin-Elmer BX (400 to 4000 cm^−1^) instrument (PerkinElmer, Boston, MA, USA). NMR spectra were recorded on a Bruker Avance DMX400 FT-NMR spectrometer instrument (Bruker, Billerica, MA, USA) operating at 400 MHz for ^1^H and 100 MHz for ^13^C at room temperature using deuterated methanol. Signals were referred to the internal standard tetramethylsilane (TMS). Chemical shifts are reported in δ units and coupling constants (*J*) in Hz. HR-ESIMS was recorded on an LTQ-XL Mass Spectrometer (Thermo Finnigan, Bremen, Germany); electrospray ionization (ESI) negative mode; capillary temperature 330 °C; source temperature 250 °C; sheath gas flow 50 arb (arb stands for arbitrary units), auxiliary gas 10 arb; source voltage 3 kV; capillary voltage −16 V; normalized collision energy 35%; 4 scan events starting with full scan 50 to 1500 amu (amu stands for atomic mass unit). Scan Events 2 to 4 were dependent scans taking the most intensive ion from the precursor spectrum TLC: silica gel 60 F (Merck KGaA, Darmstadt, Germany). Preparative TLC: silica gel 60 F self-made plates (Merck); spots and bands were viewed under UV light (254 and 366 nm). A mixture of CHCl_3_ and MeOH in a ratio of 4:1 was used as a solvent system for both analytical and preparative TLC.

### 3.2. Plant Material

The leaf latex of *A. pulcherrima* was collected in October 2011 from Debrelibanos Monastry around Amanuel Church, 100 km northwest of Addis Ababa, Central Ethiopia. The plant was authenticated at the National Herbarium, Department of Biology (courtesy of Prof. Sebsebe Demissew), Addis Ababa University, where a voucher specimen was deposited with collection number TT 001.

### 3.3. Experimental Animals

Swiss albino mice of either sex weighing 21 to 30 g and age 6 to 7 weeks were obtained from Aklilu Lemma Institute of Pathobiology animal house, Addis Ababa. All animals were outbred strains, but had undergone several generations of inbreeding. They were not subjected to any drug or test substance investigation prior to the experiments, were bred selectively in the ratio of 1 male to 3 females and kept in a 12 h light/dark cycle and at room temperature, provided with commercial pelleted ration and clean water ad libitum. The animals were cared for feeding, watering, housing, analgesia and any health problems at all time points and especially during experimental periods. All animals were kept at room temperature in an air-conditioned room and were allowed to acclimatize for one week before the study. All of the experiments were conducted in accordance with the internationally-accepted laboratory animal use, care and guidelines [36] and approved by the institutional Ethics Review Board of the School of Pharmacy, Addis Ababa University. 

### 3.4. Parasite and Preparation of Inoculum

The *Plasmodium berghei* ANKA strain (chloroquine sensitive) was obtained from the animal house of Biology Department, College of Natural Sciences, Addis Ababa University. The parasite was subsequently maintained in the laboratory by serial blood passage from a donor mouse to normal mice trough intraperitoneal (IP) inoculation on a weekly basis. Parasitemia was assessed by thin blood films made by collecting blood from the cut tip of the tail, stained with Giemsa stain and viewed under a photo microscope. For passaging the parasite into the test animals, 0.2 mL of blood were collected from the auxiliary plexus of the veins of one of the donor rats (parasitemia > 35%). The blood was diluted with 5 mL of physiological saline, to give 2 × 10^7^ parasitized red blood cells (PRBC) in an injection volume of 0.2 mL (IP) [37].

### 3.5. Extraction of the Latex

The leaf latex of *A. pulcherrima* was collected by cutting the leaves transversely near the base and allowing the yellow sap to come down in a plate. The sap was then left in open air for three days to allow evaporation of water, which yielded a dark purple solid (latex). Analytical TLCs of the fresh latex and that of the air-dried samples were compared before proceeding with the isolation procedures. 

### 3.6. Isolation of Compounds

The latex (125 mg) was initially dissolved in methanol and directly applied to preparative thin layer chromatographic (PTLC) plates over silica gel (Merck, G 6; 20 cm × 20 cm). The chromatograms were then developed in a solvent system of chloroform and methanol mixture (4:1) and visualized under UV light of 254 and 366 nm. The isolated compounds were further purified by repeated 0.25 mm-thick chromatographic plates. The bands were scraped off, washed with methanol and ethyl acetate (1:1) and filtered to yield two yellow amorphous compounds, **1** (66.3 mg) and **2** (51.2 mg), with *R_f_* values of 0.54 and 0.34 (CHCl_3_/MeOH; 4:1), respectively.

*Nataloin* (**1**): Yield 53.04% (*w*/*w*, from leaf latex); a yellow amorphous powder; −ve (−ve stands for negative) HRESI-MS *m*/*z*: 417.11879 [M − H]^−^ (calc. for C_21_H_22_O_9_: 417.118560 [M − H]^−^; UV λ_max_ nm: 267, 275, 309; ^1^H-NMR δ ppm: 2.38 (3H, *s*, H_3_-15), 2.91–3.57 (6H, *m*, H-2′ to H-6′), 3.27 (1H, *brs*, H-1′), 4.46 (1H, *brs*, H-10), 6.68 (1H, *brs*, H-4), 6.87 (1H, *brs*, H-2), 6.92 (1H, *d*, *J* = 8.0 Hz, H-6), 7.04 (1H, *d*, *J* = 8.0 Hz, H-5), 7.91 (1H, *brs*, 7-OH), 11.91 (1H, *brs*, 8-OH), 11.92 (1H, *brs*, 1-OH); ^13^C-NMR δ ppm: 20.8 (C-15), 43.6 (C-10), 61.9 (C-6′), 70.5 (C-4′), 70.6 (C-2′), 78.6 (C-1′), 80.1 (C-3′), 85.3 (C-5′), 115.2 (C-4), 117.5 (C-14), 117.6 (C-5), 119.3 (C-11), 119.6 (C-2), 120.0 (C-6), 131.2 (C-12), 144.3 (C-13), 145.9 (C-7), 147.7 (C-8), 149.7 (C-3), 161.6 (C-1), 194.5 (C-9) (see supporting information). 

*7-Hydroxyaloin* (**2**): Yield 40.96% (*w*/*w*, from leaf latex); a pale yellow amorphous substance; −ve HRESI-MS *m*/*z*: 433.11357 [M − H]^−^ (calc. for C_21_H_22_O_10_: 433.113475 [M − H]^−^); UV λ_max_ nm: 266, 274, 307; ^1^H-NMR δ ppm: 2.90–3.62 (6H, *m*, H-2′ to H-6′), 3.15 (1H, *brs*, H-1′), 4.51 (1H, *brs*, H-10), 4.66 (2H, *brs*, H_2_-15), 6.88 (1H, *brs*, H-2), 7.04 (1H, *brs*, H-4), 7.06 (1H, *d*, *J* = 7.6 Hz, H-6), 7.34 (1H, *d*, *J* = 7.6 Hz, H-5), 7.95 (1H, *brs*, 7-OH), 11.96 (1H, *brs*, 8-OH), 11.97 (1H, *brs*, 1-OH); ^13^C-NMR δ ppm: 43.83 (C-10), 61.8 (C-6′), 63.1 (C-15), 70.4 (C-4′), 70.5 (C-2′), 78.6 (C-1′), 80.1 (C-3′), 85.1 (C-5′), 112.7 (C-4), 116.2 (C-14), 116.4 (C-5), 117.6 (C-11), 119.4 (C-6), 120.1 (C-2), 131.2 (C-12), 142.6 (C-13), 144.3 (C-7), 145.1 (C-8), 150.9 (C-3), 161.7 (C-1), 194.6 (C-9) (see supporting information).

### 3.7. Acute Oral Toxicity Test

Twenty-four female mice weighing 23 to 25 g were randomly divided into 4 groups of 6 mice per cage. The animals were physically active and were consuming food and water in a regular way. Before the administration of a single dose of the latex and the compounds, the mice were fasted for 2 h [38,39]. Animals of Group I, Group II and Group III were given the latex, Compound **1** and Compound **2**, respectively. The test substances were dissolved in distilled H_2_O and administered orally at a concentration of 2 g/kg. The mice in Group IV were provided with 5 g/kg latex dissolved in distilled water after following the first three groups for 14 days. Animals were observed for gross changes such as loss of appetite, hair erection, lacrimation, tremors, convulsions, salivation, diarrhea, mortality and other signs of overt toxicity [38].

### 3.8. In Vivo Antimalarial Test

The standard 4-day suppressive method as described by Peters et al. [40] was followed to evaluate the blood schizonticidal action against *P. berghei*. Donor albino mice previously infected with chloroquine-sensitive *P*. *berghei* and with a rising parasitemia of >35% as determined using thin blood film, were sacrificed and the blood collected using an EDTA bottle. Twenty-five male Swiss albino mice weighing 21 to 28 g were infected with 10^6^ *P. berghei* and randomly divided into five groups of five mice per group. The first 3 groups were test groups and the remaining two control groups (each for chloroquine as a standard drug and physiological saline as a negative control). The latex and isolated compounds were prepared in three different doses (100, 200 and 400 mg/kg of body weight) and chloroquine at 25 mg/kg each dissolved in 0.5 mL distilled H_2_O. Each mouse was administered with 0.2 mL latex, isolated compounds or standard. The test substances or standard drug were administered orally by using an oral gavage as a single dose per day. Treatment was started 3 h after infection on Day 0 and was then continued daily for four days (from Day 0 up to Day 3). The mice were maintained in the animal house on a commercial diet and water ad libitum [41,42]. On the fifth day (D4) of the test, thin blood smears were prepared using well-labelled and properly-cleaned slides. The blood sample was collected from the tail snip of each mouse, and thin blood smears were prepared on microscopic slides (Sail Brand, China), fixed with methanol and stained with 10% Geimsa at pH 7.2 for 20 min. The parasitemia level was determined by counting the number of parasitized erythrocytes out of four random fields under the microscope (Olympus 6V20WHA2, Tokyo, Japan) with an oil immersion objective of 100× magnification power. The average percent parasitemia and suppression were calculated by using the following formula [41,42,43], where NC stands for the negative control. 

(1) $$\%\;\mathrm{Parasitaemia}\;=\frac{\mathrm{Number}\;\mathrm{of}\;\mathrm{parasitized}\;\mathrm{RBC}}{\mathrm{Total}\;\mathrm{number}\;\mathrm{of}\;\mathrm{RBC}\;\mathrm{count}}\times \;100,$$

(2) $$\%\;\mathrm{Suppression}\;=\frac{\mathrm{Mean}\;\mathrm{parasitaemia}\;\mathrm{of}\;\mathrm{NC}-\mathrm{Mean}\;\mathrm{parasitaemia}\;\mathrm{of}\;\mathrm{treated}\;\mathrm{group}}{\mathrm{Mean}\;\mathrm{parasitaemia}\;\mathrm{of}\;\mathrm{NC}}\times \;100,$$

Similar procedures were followed for the determination of the antimalarial activity of the isolated compounds. Body weights of the mice were also determined to observe the weight loss prevention effect of the latex or the isolated compounds that commonly occurs with increasing parasitemia in infected mice. Weights were taken on Day 0 (D0) and Day 5 (D4)

### 3.9. Data Analysis

The data are expressed as the mean ± the standard error of the mean (SEM). The differences between means of the measured parameters were compared using one-way analysis of variance (ANOVA) using the SPSS Windows Version 16.0 statistical package followed by a post-hoc test (Tukey method) multiple comparison and paired sample *t*-test (2-tailed). The *p*-values < 0.05 were regarded as significant. 

## 4. Conclusions

The present study aimed at identifying active antimalarial compounds from indigenous plants that may be developed into more effective and affordable drugs. From this study, it can be concluded that the leaf latex of *A. pulcherrima* possesses genuine in vivo antimalarial activity in mice infected with the rodent parasite *P. berghei*. Although neither the latex nor the isolated compounds were as active as chloroquine, the study provided scientific evidence for traditional claim of the plant for the treatment of malaria. More importantly, the activity of the anthrone *C*-glycosides nataloin (**1**) and 7-hydroxyaloin (**2**) along with their relative margin of safety merit the use of these compounds as leads for the development of safer, more potent and cost-effective alternative drugs for the treatment of malaria. However, further studies directed towards the curative effect of the test substances, as well as in-depth pharmacokinetic and pharmacodynamic studies are needed, so as to elucidate their mechanism of action. 

## Figures and Tables

**Table 1:** Percentage suppression and mean survival time of *Plasmodium berghei*-infected mice after treatment with the leaf latex of *Aloe pulcherrima*, nataloin (**1**) and 7-hydroxyaloin (**2**).

Test Substances	Dose (mg/kg/day)	% Parasitemia ± SEM	% Suppression	Survival Time (in Days) ± SEM
Distilled water	0.5 mL	61.84 ± 5.13	-	6.0 ± 0.3
Latex	100	57.26 ± 6.12 ^b^	7.4	6.4 ± 0.6 ^b^
	200	38.23 ± 3.86 ^a^	38.2	7.6 ± 0.4 ^a^
	400	39.54 ± 9.57 ^a^	36.1	6.8 ± 0.2 ^a^
Nataloin (**1**)	100	49.92 ± 6.53 ^a^	19.3	6.8 ± 0.2 ^a^
	200	36.83 ± 9.9 ^a^	40.4	6.8 ± 0.5 ^a^
	400	49.33 ± 5.02 ^a^	20.2	6.8 ± 0.2 ^a^
7-Hdroxyaloin (**2**)	100	57.37 ± 6.46 ^b^	7.2	6.4 ± 0.4 ^b^
	200	27.07 ± 9.47 ^a^	56.2	6.8 ± 0.5 ^a^
	400	56.48 ± 9.94 ^b^	8.7	6.2 ± 0.5 ^b^
Chloroquine	25	0.00	100.0	ND

Values are presented as M (Mean) ± SEM; *n* = 5; ^a^ (*p* < 0.05) compared to the negative control in the same column; ^b^ (*p* > 0.05) compared to the negative control in the same column; ND = no death within the follow-up at 28 days.

**Table 2:** Body weight of *Plasmodium berghei*-infected mice after the administration of leaf latex of *Aloe pulcherrima*, nataloin (**1**) and 7-hydroxyaloin (**2**).

Test Substance	Dose (mg/kg/day)	Wt D0 ± SEM	Wt D4 ± SEM	Mean Difference
Distilled water	0.5 mL	22.24 ± 0.39	21.40 ± 0.24	−0.84 (−3.8)
Latex	100	22.90 ± 0.51	23.90 ± 0.64	1.00 (4.4)
	200	23.00 ± 0.88	21.10 ± 0.64	−1.9 (−8.3)
	400	21.50 ± 0.35	18.90 ± 0.24	−2.6 (−11.9)
Nataloin (**1**)	100	23.74 ± 0.15	23.06 ± 0.45	−0.68 (−2.9)
	200	23.70 ± 0.18	23.18 ± 0.54	−0.52 (−2.2)
	400	22.16 ± 0.14	22.12 ± 0.11	−0.04 (−0.2)
7-Hdroxyaloin (**2**)	100	22.0 ± 0.45	21.28 ± 0.88	−0.72 (−3.3)
	200	22.30 ± 0.44	22.48 ± 0.85	0.18 (0.8)
	400	20.80 ± 0.37	20.16 ± 0.84	−0.64 (−3.1)
Chloroquine	25	24.80 ± 0.20	26.16 ± 0.81	1.36 (5.5)

Values are presented as M ± SEM; *n* = 5; Wt D0: weight pre-treatment on Day 0; Wt D4: weight post-treatment on Day 5; values in parenthesis indicate % of change.

## Supplementary Materials

The following are available online at www.mdpi.com/1420-3049/21/11/1415/s1. The IR, NMR and HR-MS spectra of Compounds **1** and **2** are included in the Supplementary Data.
