# Supplementary material for: Antimalarial Activity of the Chemical Constituents of the Leaf Latex of Aloe pulcherrima Gilbert and Sebsebe
Source: Molecules. 2016 Oct 28;21(11):1415. doi: 10.3390/molecules21111415 (PMC6273959; doi:10.3390/molecules21111415)
Supplement: Supplementary file 1 [file molecules-21-01415-s001.pdf]

Supplementary Materials: Antimalarial Activity of the Chemical Constituents of the Leaf Latex of *Aloe pulcherrima* Gilbert and Sebsebe

Tekleab Teka, Daniel Bisrat, Mariamawit Yonathan Yeshak and Kaleab Asres

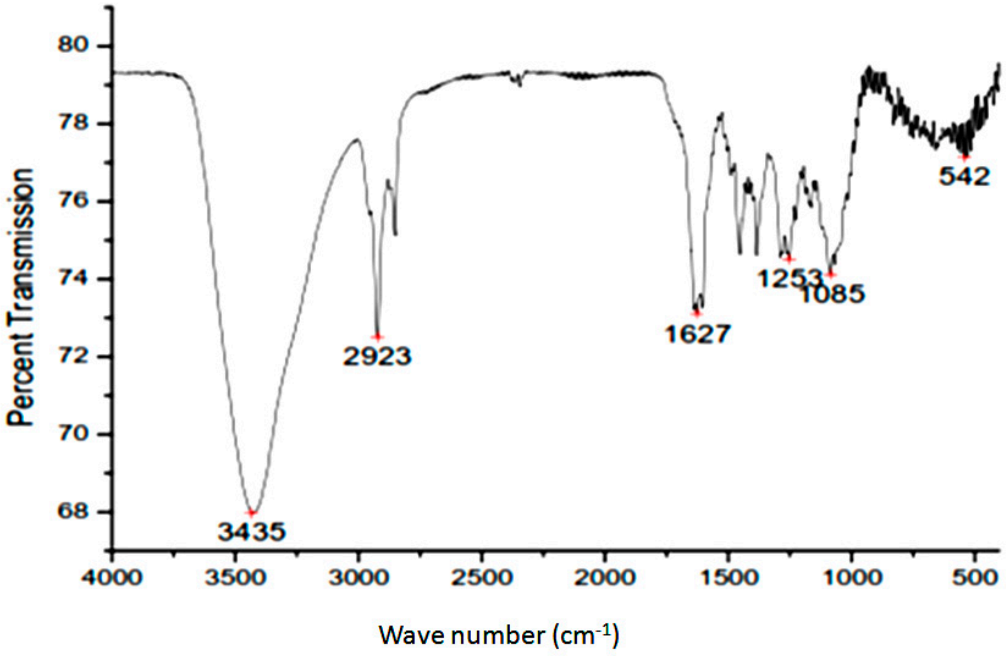

Figure S1. IR spectrum of Compound **1** (nataloin).

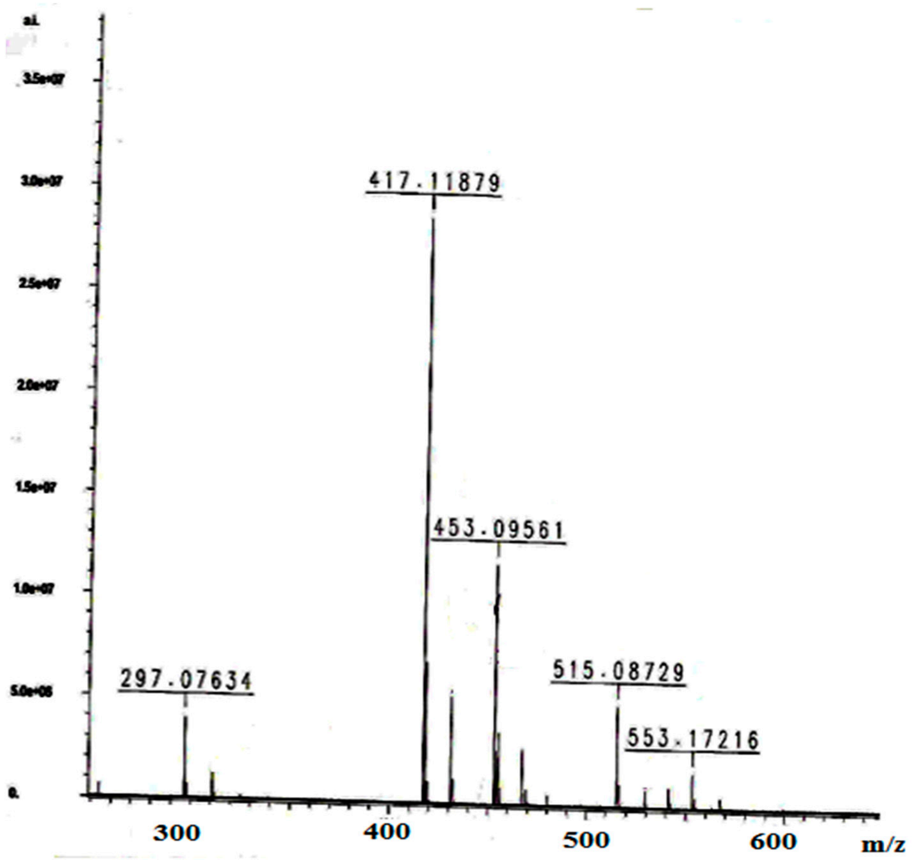

Figure S2. Negative-mode HRESI-Mass spectrum of Compound **1** (nataloin).

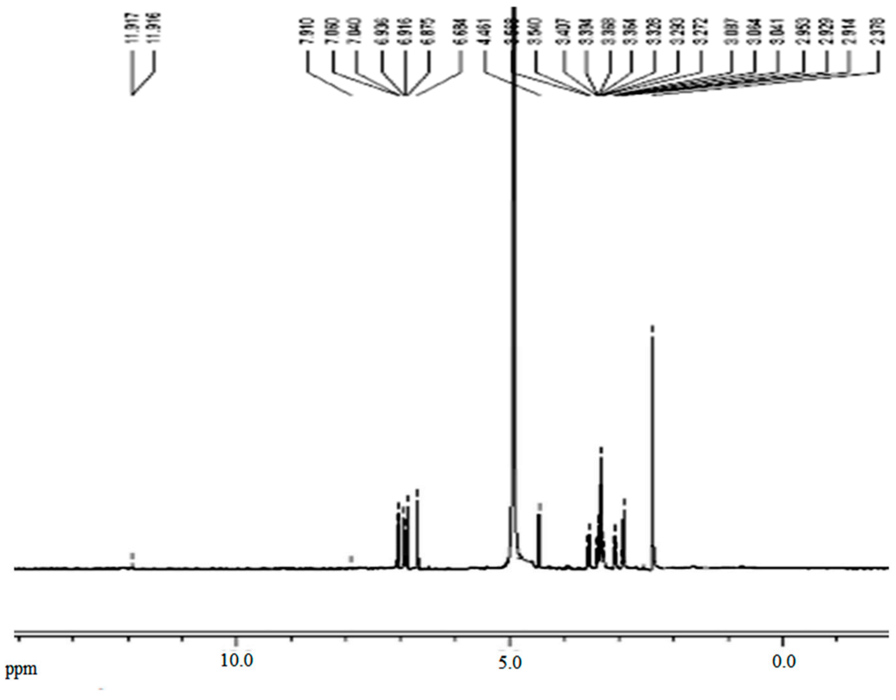

Figure S3. ^{1}H-NMR spectrum of Compound **1** (nataloin).

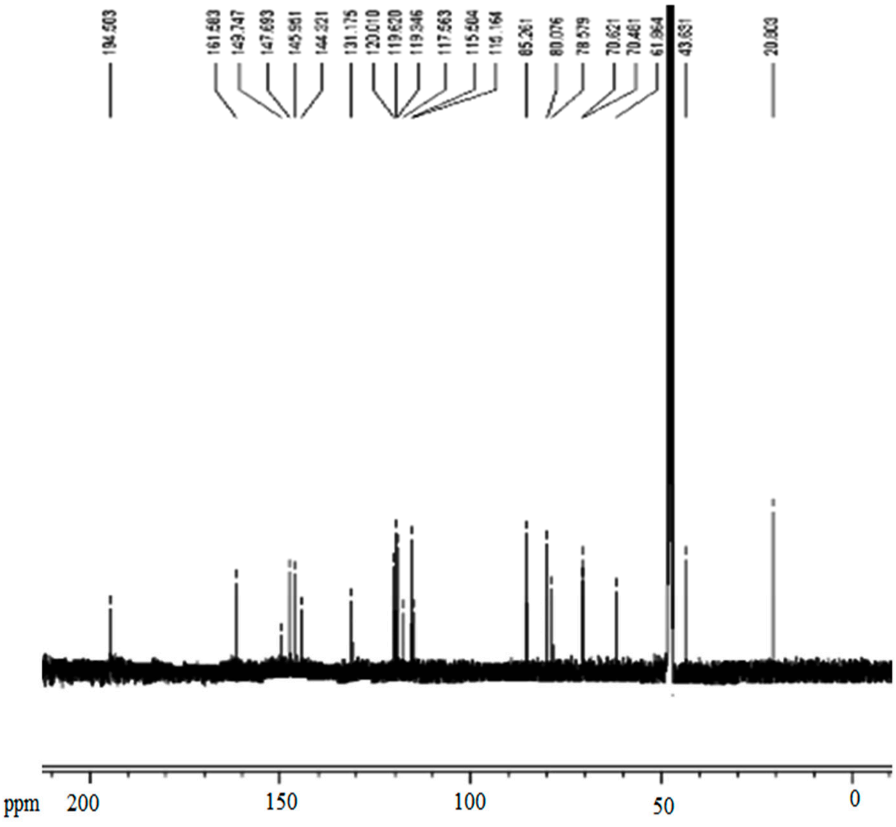

Figure S4. ^{13}C-NMR spectrum of Compound **1** (nataloin).

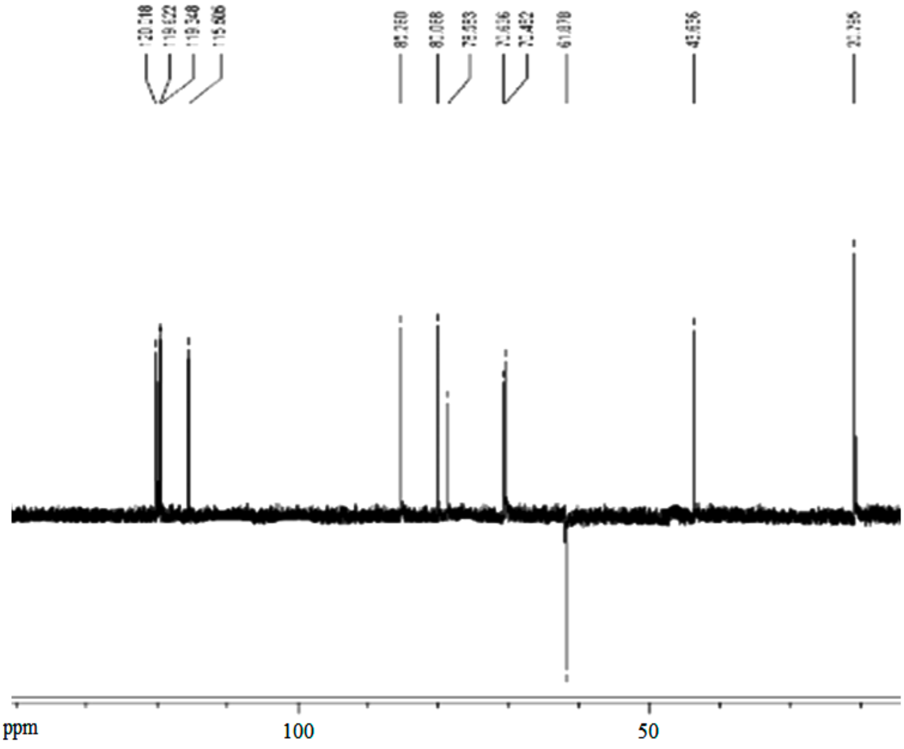

Figure S5. DEPT-135 spectrum of Compound **1** (nataloin).

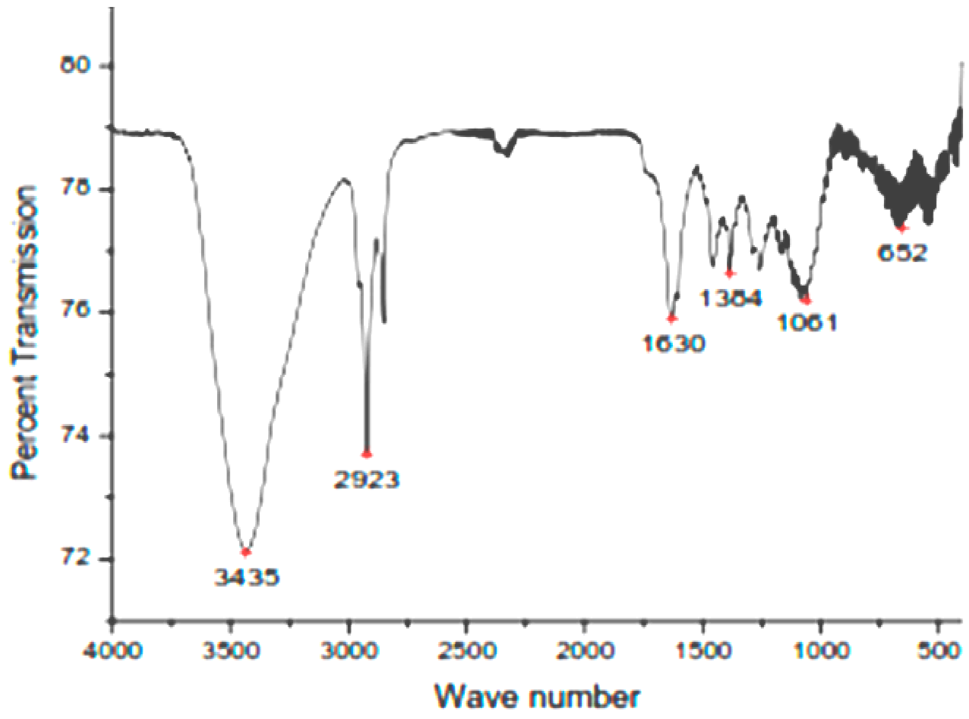

Figure S6. IR spectrum of Compound **2** (7-hydroxyaloin).

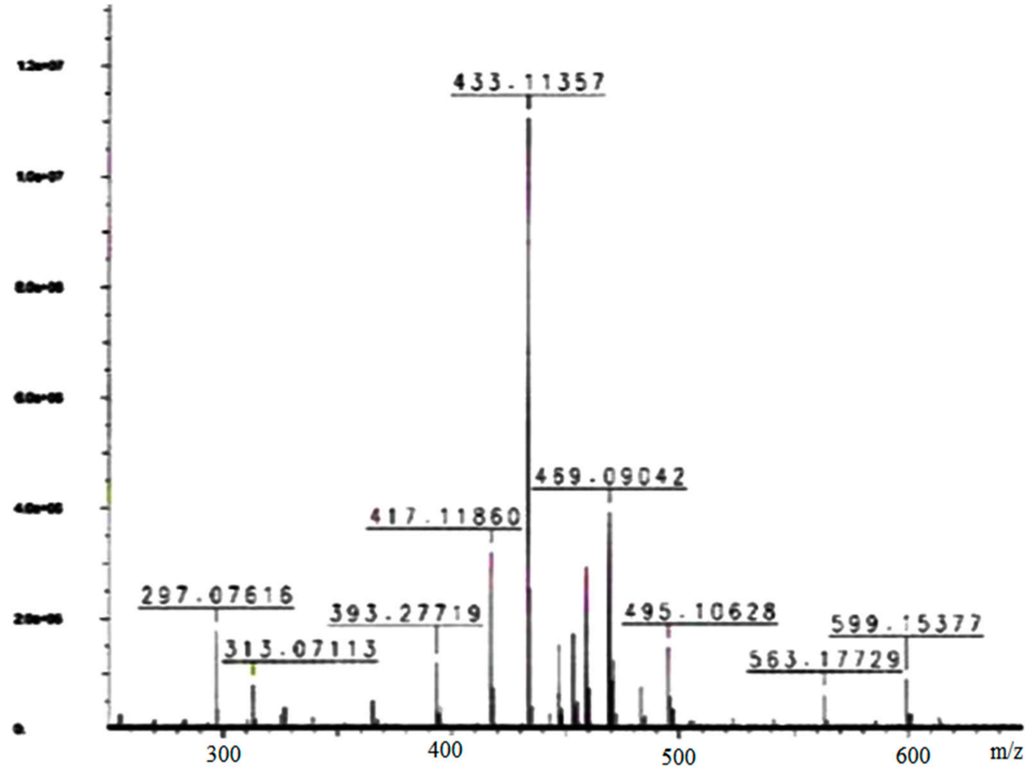

Figure S7. Negative-mode HRESI-Mass spectrum of Compound **2** (7-hydroxyaloin).

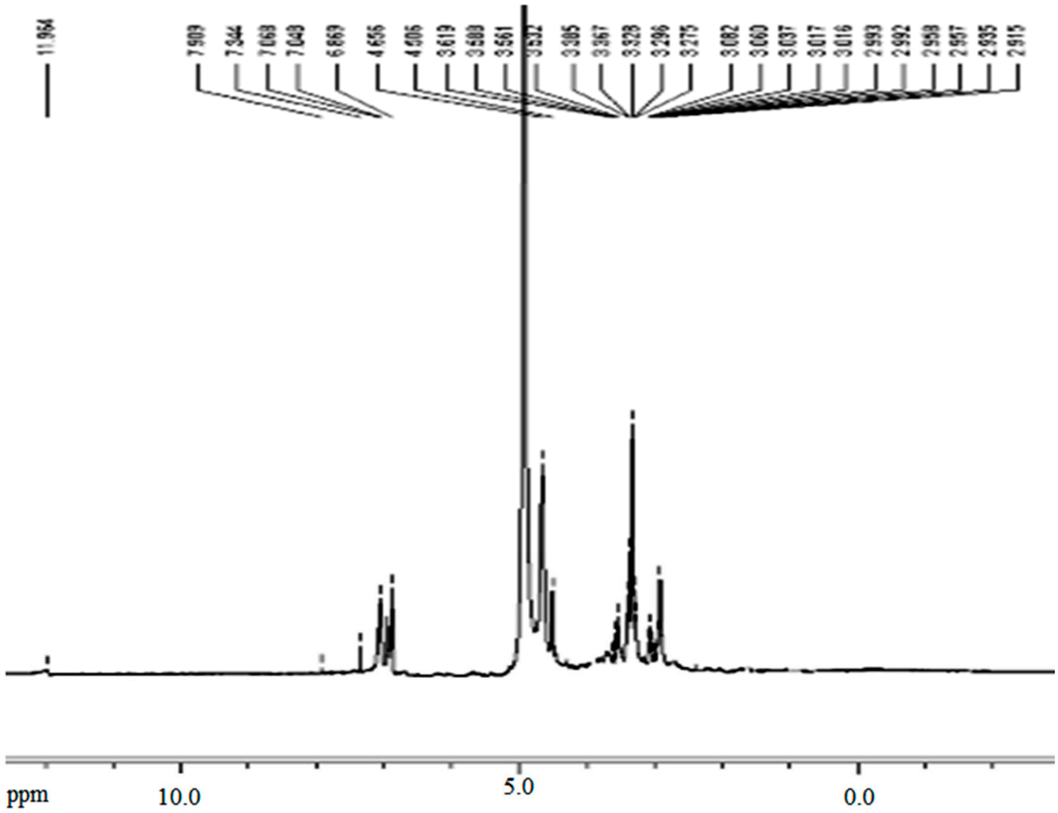

Figure S8. ^{1}H-NMR spectrum of Compound **2** (7-hydroxyaloin).

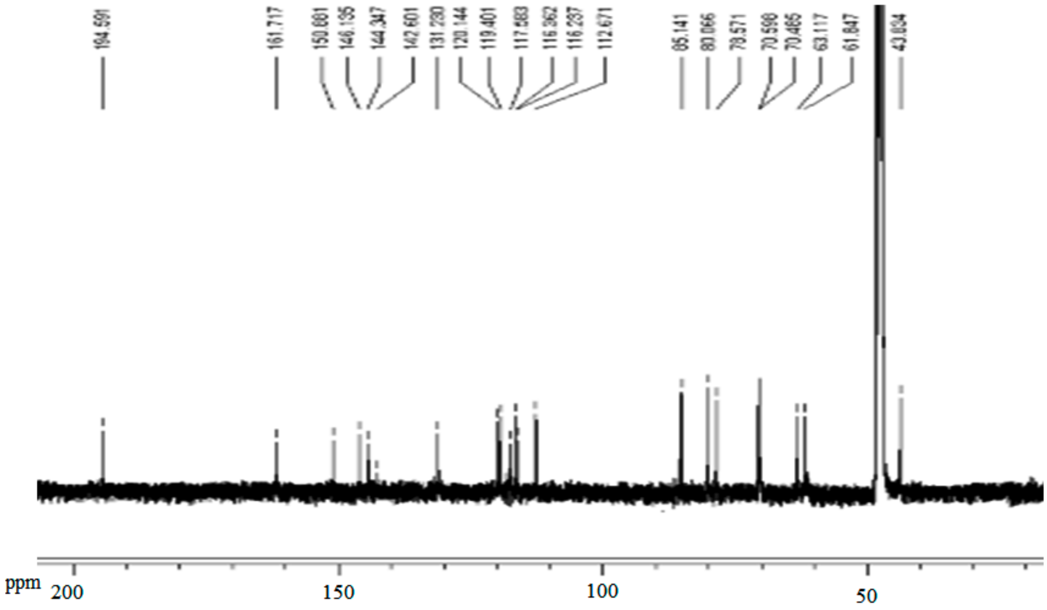

Figure S9. ^{13}C-NMR spectrum of Compound **2** (7-hydroxyaloin).

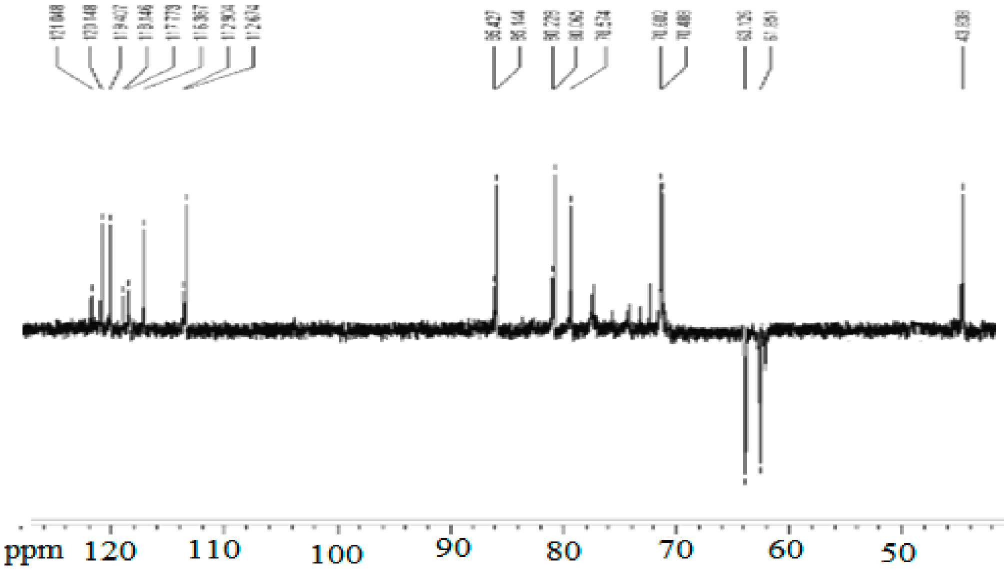

Figure S10. DEPT-135 spectrum of Compound **2** (7-hydroxyaloin).
